# Supplementary material for: Fluorescent Anti-CEA Nanobody for Rapid Tumor-Targeting and Imaging in Mouse Models of Pancreatic Cancer
Source: Biomolecules. 2022 May 16;12(5):711. doi: 10.3390/biom12050711 (PMC9138244; doi:10.3390/biom12050711)
Supplement: Supplementary file 1 [file biomolecules-12-00711-s001.zip › biomolecules-1702018-supplementary.pdf]

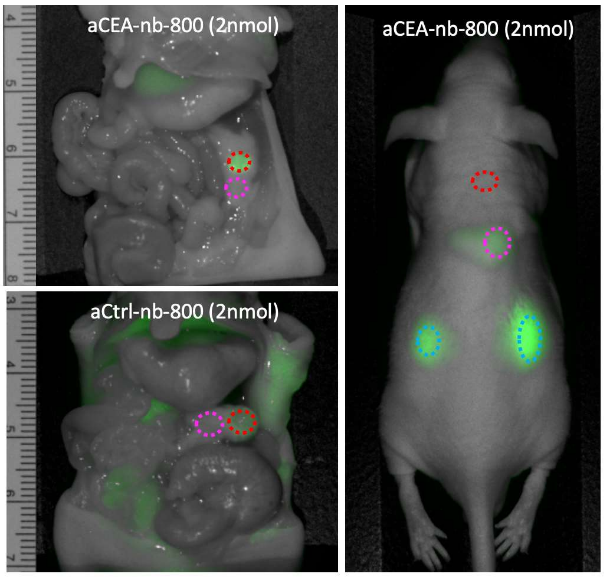

Figure S1. Sample regions of interest in orthotopic and subcutaneous patient derived xenograft mouse models
